# Supplementary material for: SCAMPP+FastTree: improving scalability for likelihood-based phylogenetic placement
Source: Bioinform Adv. 2023 Jan 30;3(1):vbad008. doi: 10.1093/bioadv/vbad008 (PMC9933845; doi:10.1093/bioadv/vbad008)
Supplement: vbad008_Supplementary_Data [file vbad008_supplementary_data.pdf]

Supplementary Materials for “SCAMPP+FastTree: Improving Scalability for Likelihood-based Phylogenetic Placement”

Gillian Chu and Tandy Warnow

Department of Computer Science, University of Illinois at Urbana-Champaign

Contents

1 Additional Results **2**
- 1.1 Results on full-length query sequences . . . 2
- 1.2 Overall results . . . 3
- 1.3 Distribution of Delta Error . . . 6

2 Commands **12**
- 2.1 Delta Error . . . 12
- 2.2 Numeric Parameters . . . 12
- 2.3 Placement Methods . . . 12
  - 2.3.1 pplacer-SCAMPP-RAxML . . . 12
  - 2.3.2 pplacer-FastTree . . . 13
  - 2.3.3 pplacer-SCAMPP-FastTree . . . 13
  - 2.3.4 EPA-ng . . . 13
  - 2.3.5 APPLES-2 . . . 13

3 Additional Information About Datasets **14**
- 3.1 The nt78 simulated dataset . . . 14
- 3.2 Impact of duplicate sequences in FastTree . . . 14

4 Comparison of RAxML and FastTree 2 for Numeric Parameter Estimation **15**

List of Figures

- S1 Results on nt78 and ROSE with full-length query sequences . . . 2
- S2 Histogram of delta error on green85 . . . 6
- S3 Histogram of delta error on LTP . . . 7
- S4 Histogram of delta error on 16S.B.ALL . . . 8
- S5 Histogram of delta error on nt78, 0.10 query length . . . 9
- S6 Histogram of delta error on nt78, 0.25 query length . . . 10
- S7 Histogram of delta error on nt78, full-length queries . . . 11

List of Tables

- S1 Results on simulated datsets . . . 4
- S2 Results on biological datasets . . . 5
- S3 Dataset statistics . . . 14

1 Additional Results

1.1 Results on full-length query sequences

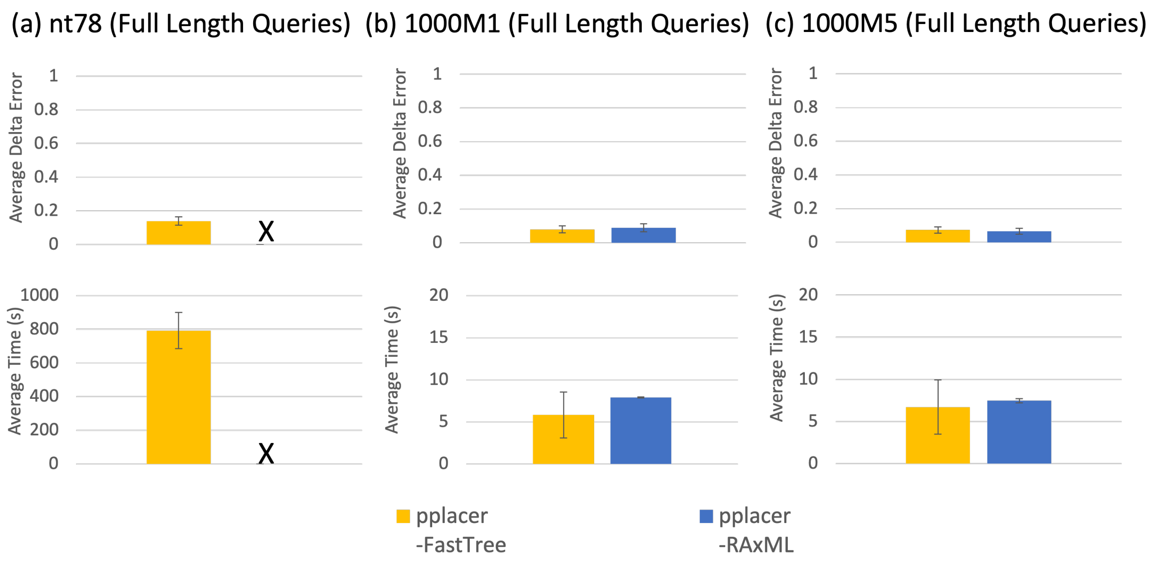

Figure S1: **(Experiment 1) Comparison of pplacer-RAxML and pplacer-FastTree on the nt78 and ROSE datasets, in placing full-length query sequences.** (a) Results on the first replicate of the nt78 dataset containing 78,132 sequences. (b) Results on the ROSE 1000M1 datasets (first 5 replicates) containing 1000 sequences. (c) Results on the ROSE 1000M5 datasets (first 5 replicates) containing 1000 sequences. The top row shows average delta error, bottom row shows runtime (each per query). The "X" indicates that pplacer-RAxML failed with an out of memory error on these experiments. Peak memory usage on the ROSE datasets is negligible (< 1GB).

1.2 Overall results

Delta Error	nt78			RNASim					ROSE 1000-Seq	
	nt78 (full)	nt78 (0.25)	nt78 (0.10)	50k (full)	50k (0.25)	100k (full)	100k (0.25)	200k (full)	1000M1	1000M5
pplacer-FastTree	**0.14**	**0.69**	**1.54**	**0.07**	**0.21**	**0.06**	**0.06**	X	**0.10**	**0.07**
pplacer-RAxML	X	X	X	X*	X*	X*	X*	X*	**0.09**	**0.07**
pplacer-SCAMPP -RAxML (2k)	**0.14**	6.90	12.51	-	-	-	-	-	-	-
pplacer-SCAMPP -FastTree (10k)	**0.14**	3.89	12.89	**0.07**	**0.22**	**0.07**	**0.14**	**0.06**	-	-
pplacer-SCAMPP -FastTree (40k)	**0.14**	0.90	3.74	**0.08**	**0.21**	**0.07**	**0.13**	**0.06**	-	-
APPLES-2	**0.22**	1.50	2.84	0.18	0.59	**0.14**	0.59	**0.10**	-	-
EPA-ng	X	X	X	X*	X*	X*	X*	X*	-	-

Runtime (s)	nt78			RNASim					ROSE 1000-Seq	
	nt78 (full)	nt78 (0.25)	nt78 (0.10)	50k (full)	50k (0.25)	100k (full)	100k (0.25)	200k (full)	1000M1	1000M5
pplacer-FastTree	760	324	248	496	202	1178	1223	X	**6**	**7**
pplacer-RAxML	X	X	X	X*	X*	X*	X*	X*	8	**7**
pplacer-SCAMPP -RAxML (2k)	26	**15**	**11**	-	-	-	-	-	-	-
pplacer-SCAMPP -FastTree (10k)	394	79	37	435	120	442	153	**457**	-	-
pplacer-SCAMPP -FastTree (40k)	1592	483	245	1775	494	881	278	1809	-	-
APPLES-2	**21**	24	25	**14**	**26**	**26**	**14**	750	-	-
EPA-ng	X	X	X	X*	X*	X*	X*	X*	-	-

Memory (GB)	nt78			RNASim					ROSE 1000-Seq	
	nt78 (full)	nt78 (0.25)	nt78 (0.10)	50k (full)	50k (0.25)	100k (full)	100k (0.25)	200k (full)	1000M1	1000M5
pplacer-FastTree	24.362	6.455	2.799	18.703	18.699	37.523	37.529	X	**0.000**	**0.000**
pplacer-RAxML	X	X	X	X*	X*	X*	X*	X*	**0.000**	**0.000**
pplacer-SCAMPP -RAxML (2k)	**0.066**	**0.021**	**0.000**	-	-	-	-	-	-	-
pplacer-SCAMPP -FastTree (10k)	3.295	0.993	0.583	3.867	1.087	**3.921**	**1.212**	4.176	-	-
pplacer-SCAMPP -FastTree (40k)	12.695	3.531	1.668	15.040	4.116	7.643	2.205	15.475	-	-
APPLES-2	**0.000**	**0.018**	**0.004**	**0.011**	**0.000**	15.242	4.188	**1.271**	-	-
EPA-ng	X	X	X	X*	X*	X*	X*	X*	-	-

Table S1: **Summary for Delta Error, Runtime, and Peak Memory Consumption on Large Backbone Trees for Simulated Data:** We provide a summary of the per-query delta error (top) and runtime (bottom) results shown across the methods on the simulated datasets with large backbone trees (we omit the ROSE datasets because they are too small). We show the query lengths in the row below the dataset. 'X' indicates the method failed to run on this dataset. 'X*' indicates these methods were not run but were expected to fail from previous studies. For instance, RNASim-50k and pplacer-RAxML were not run because they have already been shown to fail [7]. The lowest delta error for each dataset column (including all ties within 0.1) is in boldface. '-' indicates this method was not run on the corresponding dataset.

**Delta Error**	**biological**		
	**16S.B.ALL**	**LTP_s128_SSU**	**green85**
**pplacer-FastTree**	4.10	**0.46**	**0.60**
**pplacer-RAxML**	4.08	**0.48**	4.17
**pplacer-SCAMPP -RAxML (2k)**	4.21	**0.52**	2.31
**pplacer-SCAMPP -FastTree (25%)**	4.01	**0.46**	0.73
**pplacer-SCAMPP -FastTree (50%)**	4.11	**0.45**	**0.65**
**APPLES-2**	14.27	12.53	5.23
**EPA-ng**	**3.40**	0.63	2.21

**Runtime (s)**	**biological**		
	**16S.B.ALL**	**LTP_s128_SSU**	**green85**
**pplacer-FastTree**	191	92	29
**pplacer-RAxML**	240	444	36
**pplacer-SCAMPP -RAxML (2k)**	**25**	22	18
**pplacer-SCAMPP -FastTree (25%)**	242	130	46
**pplacer-SCAMPP -FastTree (50%)**	484	279	97
**APPLES-2**	196	**1**	**3**
**EPA-ng**	484	279	97

**Memory (GB)**	**biological**		
	**16S.B.ALL**	**LTP_s128_SSU**	**green85**
**pplacer-FastTree**	**0.009**	**0.004**	0.396
**pplacer-RAxML**	31.246	4.796	5.282
**pplacer-SCAMPP -RAxML (2k)**	0.176	**0.046**	2.077
**pplacer-SCAMPP -FastTree (25%)**	**0.003**	**0.001**	0.341
**pplacer-SCAMPP -FastTree (50%)**	**0.005**	**0.002**	0.700
**APPLES-2**	**0.000**	**0.000**	**0.000**
**EPA-ng**	**0.027**	**0.017**	**0.000**

Table S2: **Summary for Delta Error, Runtime, and Peak Memory Consumption on Large Backbone Trees for Biological Data:** We provide a summary of the per-query delta error (top) and runtime (bottom) results shown across the methods on the biological datasets. We show the query lengths in the row below the dataset. 'X' indicates the method failed to run on this dataset. 'X*' indicates these methods were not run but were expected to fail from previous studies. Note that 16S.B.ALL has $27,643$ sequences, LTP_s128_SSU has $12,953$ sequences and green85 has $5,088$ sequences. The lowest delta error for each dataset column (including all ties within 0.1) is in boldface.

1.3 Distribution of Delta Error

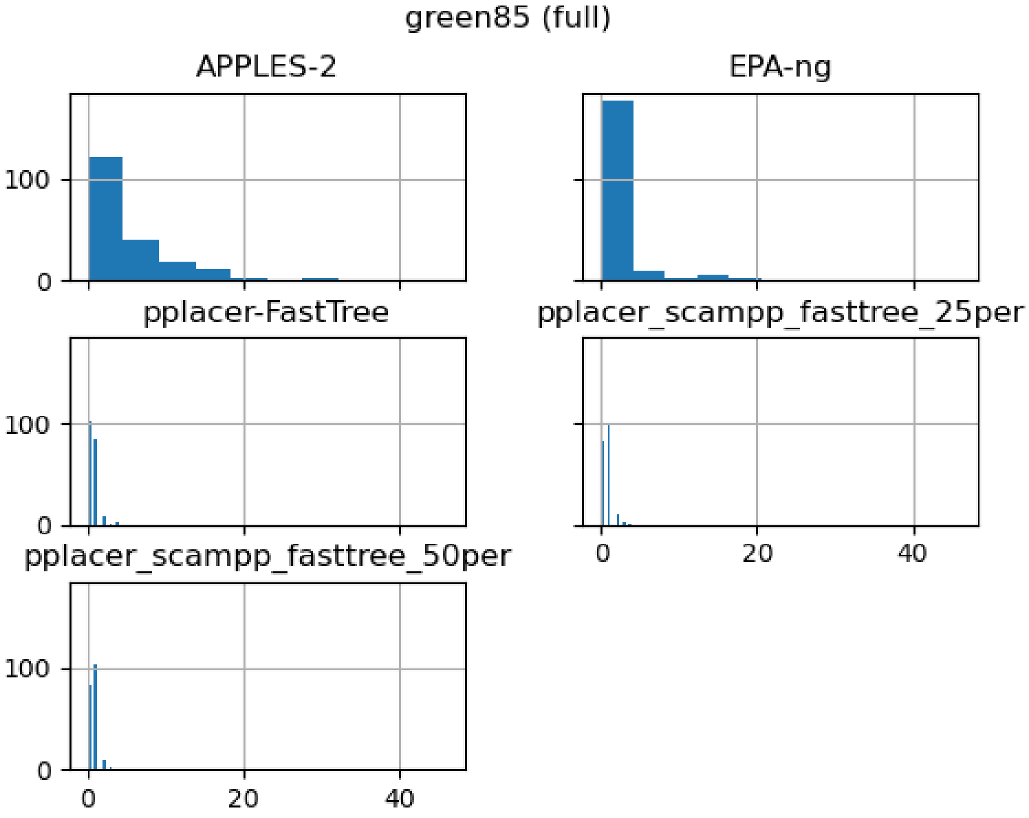

Figure S2: Histogram of delta error distribution, on the green85 biological dataset.

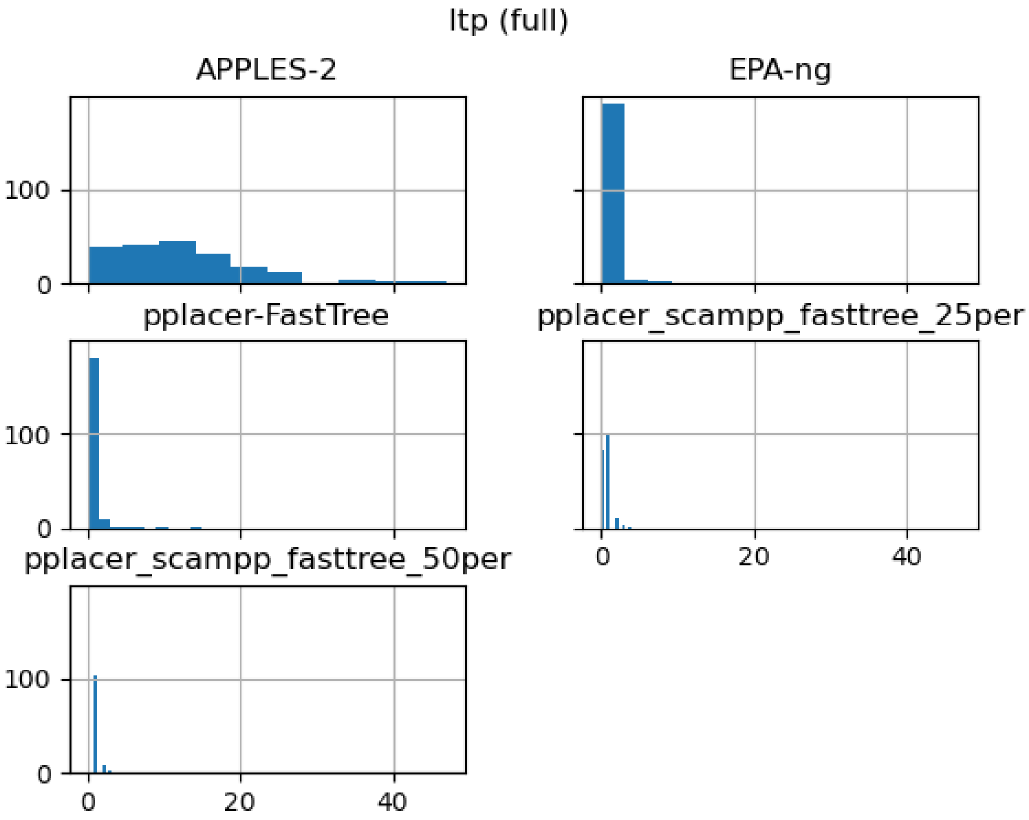

Figure S3: Histogram of delta error distribution, on the LTP biological dataset.

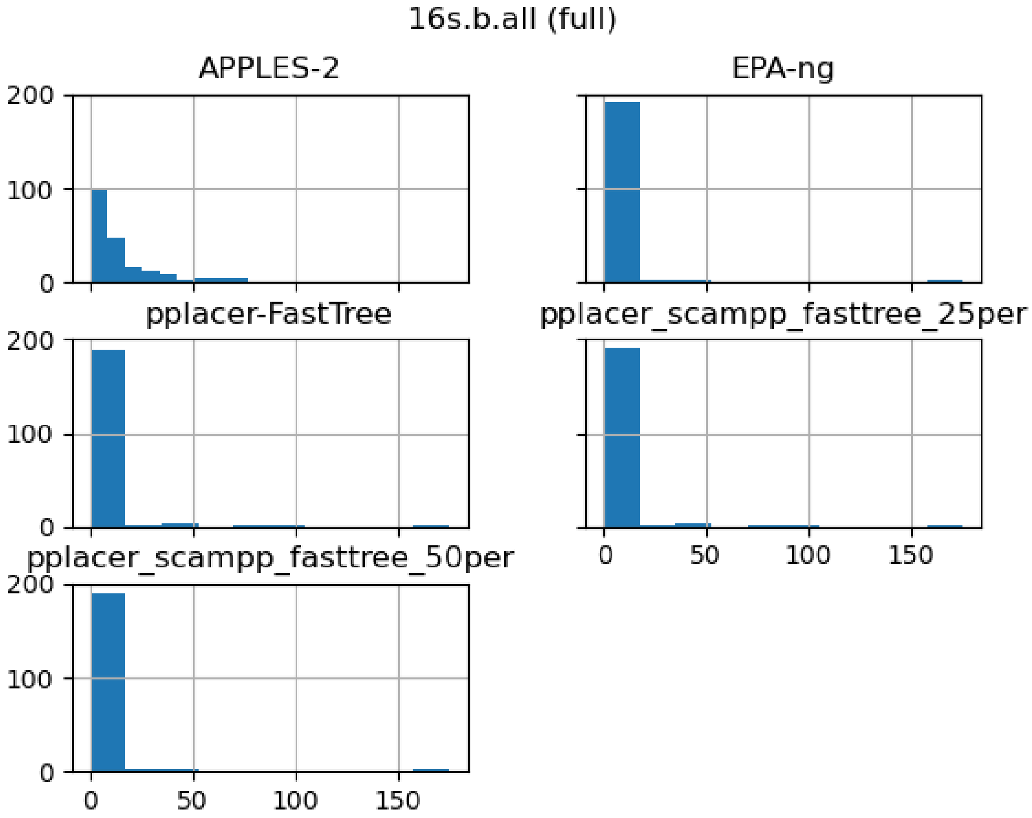

Figure S4: A histogram of delta error distribution, on the 16S.B.ALL biological dataset.

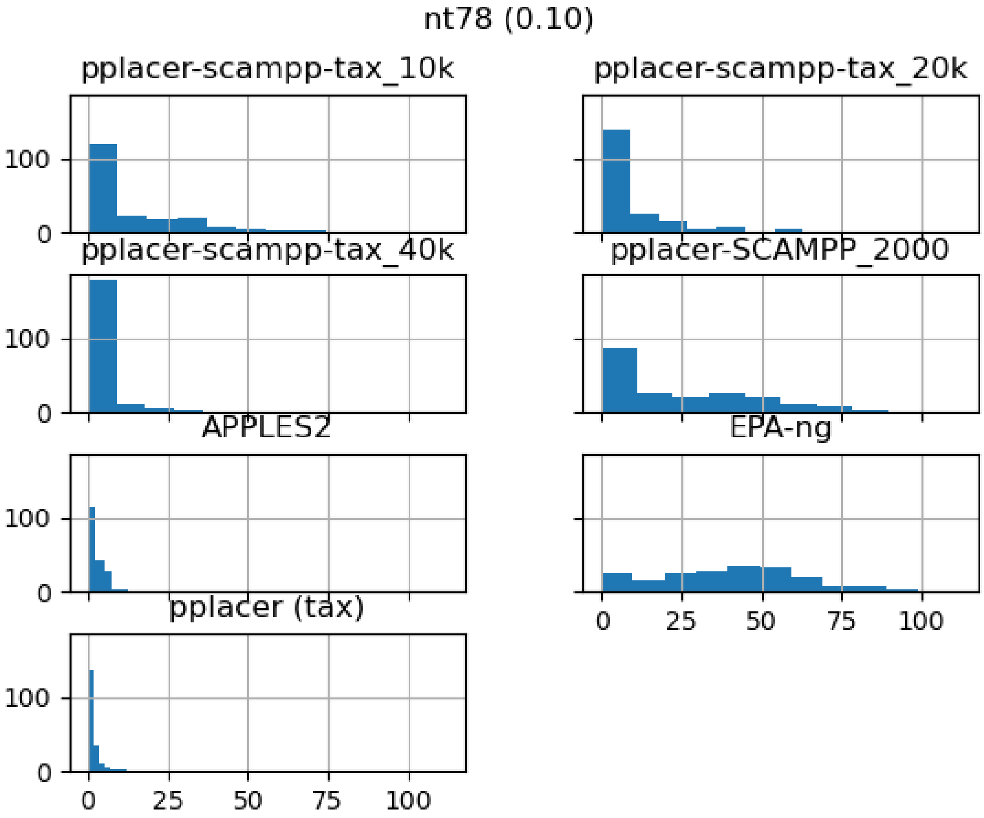

Figure S5: A histogram of delta error distribution, on the nt78 simulated dataset, evaluated at query length 0.10.

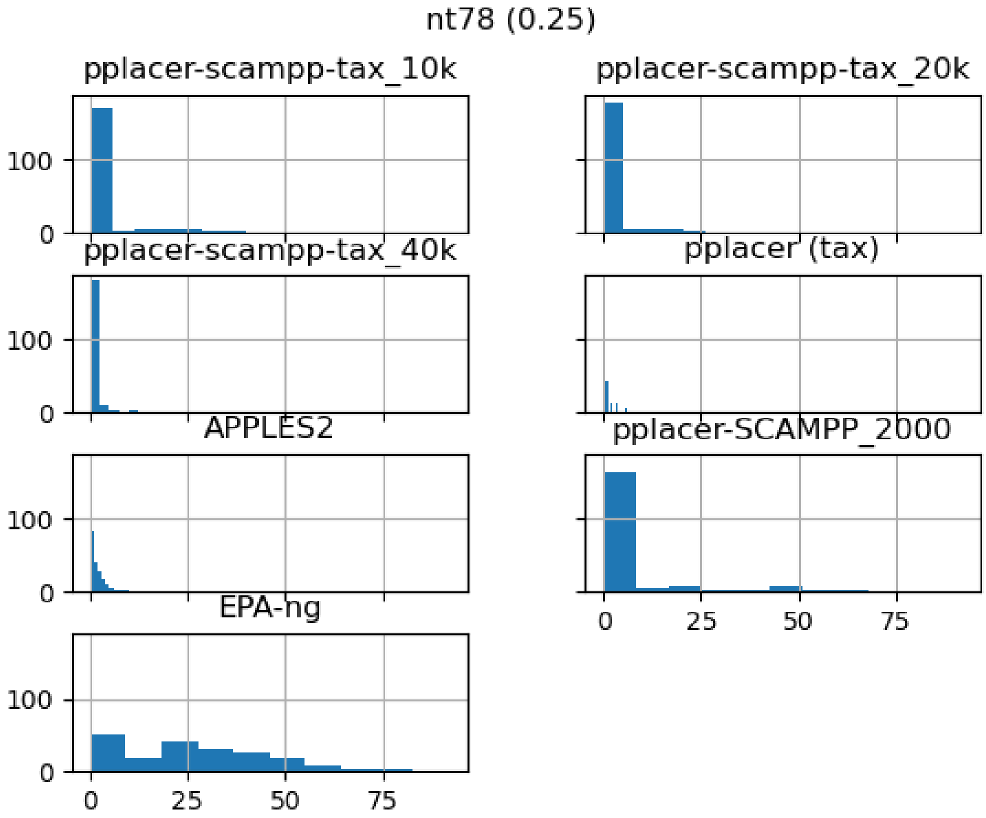

Figure S6: A histogram of delta error distribution, on the nt78 simulated dataset, evaluated at query length 0.25.

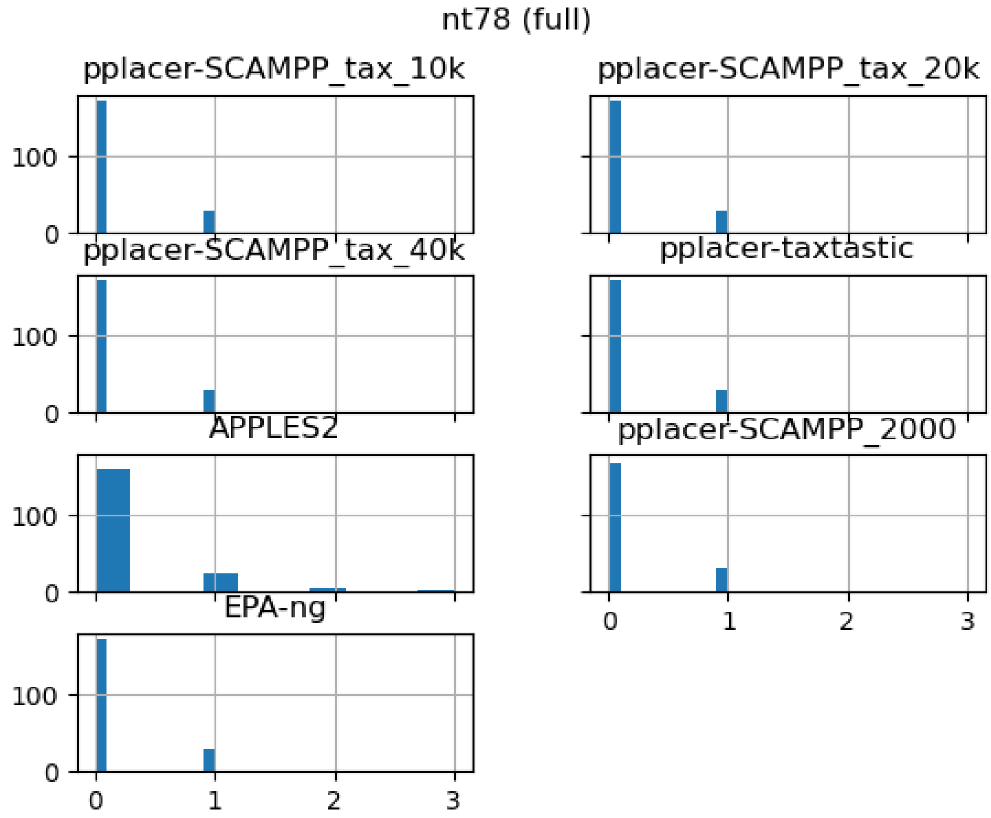

Figure S7: A histogram of delta error distribution, on the nt78 simulated dataset, evaluated for full-length query sequences.

2 Commands

2.1 Delta Error

Suppose T is the backbone tree used on leafset $\mathcal{L}$. Let q be a single query sequence and let T^* be the true tree on $\mathcal{L} \cup \{q\}$. Let P be the tree produced when the query q is added into the backbone tree T using the phylogenetic placement pipeline. For tree A and set B a subset of the leaves of A, we denote the subtree of A induced by leafset B by $A|_B$. Now for an arbitrary tree t (on whatever leafset), let $B(t)$ denote the set of bipartitions (where each edge in t defines a bipartition on its leafset). Then the delta error is the increase in the number of false negatives produced by adding q into T. Hence,

$$\Delta e(P) = |B(T^*) \backslash B(P)| - |B(T^*|_{\mathcal{L}}) \backslash B(T)|$$

The scripts to calculate delta error were adapted from published scripts [1], and also use utilities from `newick_utils`.

2.2 Numeric Parameters

Numeric parameters are estimated on a tree topology under the Generalized Time Reversible (GTR) model with gamma-distributed rates-across sites. These numeric parameters include branch lengths, the substitution rate matrix (e.g., the `4x4` Generalized Time Reversible), and gamma distribution for rates-across-sites. Each placement method specifies a preferred software for producing these estimated parameters. In this paper we used existing numeric parameters where possible and estimated using `RAxML` and `FastTree` if necessary. We provide relevant commands below.

For `pplacer-SCAMPP-FastTree`, we use published `FastTree-2`-estimated numeric parameters (e.g., branch lengths, substitution rate matrix and gamma distribution) when possible; here there are no published numeric parameters we compute all three numeric parameters using `FastTree-2` with the following command:

- `FastTreeMP -nosupport -gtr -gamma -nt -log reestimated.fasttree.log -intree intree.fasta < alignment.fasta > reestimated.fasttree.tree`

For `pplacer-SCAMPP-RAxML`, we use published numeric parameters (e.g., branch lengths, substitution rate matrix and gamma distribution) [7] for all datasets other than the `ROSE` datasets, which had not been previously evaluated in this context. We provide the `RAxML` command used to estimate all these numeric parameters on the `ROSE` datasets below:

- `RAxML-7.2.8-ALPHA/raxmlHPC-PTHREADS -f e -g reference.nwk -m GTRGAMMA -s alignment.phylip -n outname -p 1984 -T 16 -w outdir/`

For all other datasets, `RAxML 7` was used to estimate all three numeric parameters for `pplacer-SCAMPP-RAxML`, with one exception. On the RNASim dataset, we used published `FastTree-2`-estimated branch lengths [7], and `RAxML 7`-estimated substitution rate matrix and gamma distribution. We use the published substitution rate matrix and gamma distribution on the `RNASim-200k` dataset [1] for all smaller subsets of the RNASim dataset, as all subsets of the RNASim dataset come from the same million-sequence tree so we expect the substitution rate matrix and gamma distribution estimated on the RNASim-100k dataset to be quite similar to the numeric parameters for RNASim-200k.

2.3 Placement Methods

2.3.1 pplacer-SCAMPP-RAxML

We used pplacer-SCAMPP-RAxML (v2.0.0, Accessed: May 10, 2022), which can be found on github at https://github.com/chry04/PLUSplacer. We used estimated branch lengths and all other numeric

parameters from `RAxML 7` where possible (i.e., with the exception for RNASim, detailed in previous section). "jobID" is only necessary if running several jobs at once.

- `python pplacer-SCAMPP.py -i tree_info.log -t backbone_pp.tree -d out_dir/ -a full_msa.aln -b 2000 -n jobID`

2.3.2 pplacer-FastTree

We used estimated branch lengths and all other numeric parameters from `FastTree-2`.

To run `pplacer` with `FastTree` numeric parameters, we can use the `taxtastic` software to reformat the `FastTree` info files. This builds a "reference package" which consists of a backbone alignment, corresponding phylogeny and estimated numeric parameters on that phylogeny.

- `taxtastic/taxtastic-env/bin/taxit create -P ng-my.refpkg -l name --aln-fast ref.fa --tree-file backbone_pp.tree --tree-stats reestimated.fasttree.log`

The new version of `pplacer-FastTree` (using the speedup in placing fragmentary query sequences implemented on May 16, 2022) was run for results on `RNASim-100k` and `RNASim-200k`. For all other datasets and results on fragmentary query sequences, the version of `pplacer-FastTree` not implementing this speedup on fragmentary query sequences was used.

2.3.3 pplacer-SCAMPP-FastTree

We used estimated branch lengths and all other numeric parameters from `FastTree-2`. This method can be found on github at https://github.com/gillichu/PLUSplacer-taxtastic.

- `python pplacer-tax-SCAMPP.py -i reestimated.fasttree.log -t backbone_pp.tree -d outdir -q query.fa -b 2000 -o jplace_name -a ref.fa -r ref.fa -n 1563`

2.3.4 EPA-ng

We estimated branch lengths and all other numeric parameters using `RAxML 8`.

- `epa-ng --ref-msa refaln --tree bbtree --query qaln --outdir outdir --model infofile --redo -T 16`

This method failed on several datasets, due to the following out of memory error:
`slurmstepd: error: Detected 1 oom-kill event(s) in StepId=6152434.batch.`

2.3.5 APPLES-2

We estimated branch lengths and all other numeric parameters using `FastTree-2` under minimum evolution. Please note that before running APPLES-2 we converted all U in our sequences to T, as APPLES-2 only understands the ACTG alphabet.

- `run_apples.py -t bbtree -s refaln -q ${qaln} -T 16 -o outjson -D -X -f 0.2 -b 25`

3 Additional Information About Datasets

Dataset	seqs	align length	type	p-distance average	p-distance maximum	prop gaps	reps
**nt78** [6]	78,132	1,287	simulated	.404	.639	.006	1
**RNASim-50k** [5]	50,000	1,620	simulated	.410	.618	.051	5
**RNASim-100k** [5]	100,000	1,620	simulated	.410	.618	.051	1
**RNASim-200k** [5]	200,000	1,620	simulated	.410	.618	.051	1
**ROSE 1000M1** [4]	1,000	3,895	simulated	.697	.770	.738	5
**ROSE 1000M5** [4]	1,000	1,762	simulated	.501	.607	.426	5
**green85** [3]	5,088	1,486	biological	.250	.479	.146	1
**LTP_s128_SSU** [3]	12,953	1,598	biological	.228	.468	.090	1
**16S.B.ALL** [2]	27,643	6,857	biological	.210	.769	.118	1

Table S3: Dataset Statistics: the number of sequences, alignment length, type of alignment, average and maximum p-distance (normalized Hamming distance), proportion of alignment that is gapped, and number of replicates.

3.1 The nt78 simulated dataset

The nt78 simulation protocol also has some interesting features worth considering. According to the `FastTree-2` documentation for these datasets, the internal branch lengths were "truncated to be no less than 0.001 (corresponding to roughly 1 substitution along the internal branch. To make such short branch lengths with Rose... branch lengths [were scaled] by $1,000$x (not 100x) and set MeanSubstitution = 0.00134" [6].

We use the published FastTree-2 results (tree and numeric parameters estimated under Minimum Evolution) on the `nt78` datasets [7]. Published datasets from [7] can be found at https://databank.illinois.edu/datasets/IDB-9257957.

3.2 Impact of duplicate sequences in FastTree

`FastTree` and RAxML are both used in this study to estimate numeric parameters (e.g., branch lengths) on the backbone tree. However, if the dataset has duplicate sequences (even though named differently), `FastTree` has the potential to change the topology, despite being run with a command meant only to estimate the branch lengths and tree model parameters. As a result, when necessary, we modified alignments and their associated phylogenetic trees to ensure that they did not have any duplicate sequences before running FastTree (i.e., we removed all but one of the copies).

4 Comparison of RAxML and FastTree 2 for Numeric Parameter Estimation

There are many differences between FastTree and RAxML's parameter estimation process. Our commands to both FastTree and RAxML specify to estimate numeric parameters under the discrete gamma model (specified with -gamma). By default, FastTree estimates the numeric parameters with 20 discrete rate categories with a fixed rate for each site (also known as the CAT20 approximation). In contrast, by default RAxML always uses 4 rate categories to approximate the gamma distribution of rate heterogeneity, as seen in the documentation here: https://cme.h-its.org/exelixis/resource/download/NewManual.pdf on page 11.

Our study suggests that pplacer using FastTree 2 numeric parameters can scale to larger datasets and so has fewer numerical problems, compared to pplacer using RAxML. More work is needed to understand why this is true.

References

[1] Metin Balaban, Yueyu Jiang, Daniel Roush, Qiyun Zhu, and Siavash Mirarab. Fast and accurate distance-based phylogenetic placement using divide and conquer. *Molecular Ecology Resources*, 22(3):1213–1227, 2022.

[2] Jamie J Cannone, Sankar Subramanian, Murray N Schnare, James R Collett, Lisa M D'Souza, Yushi Du, Brian Feng, Nan Lin, Lakshmi V Madabusi, Kirsten M Müller, et al. The comparative RNA web (CRW) site: an online database of comparative sequence and structure information for ribosomal, intron, and other RNAs. *BMC bioinformatics*, 3(1):1–31, 2002.

[3] Benjamin Linard, Nikolai Romashchenko, Fabio Pardi, and Eric Rivals. PEWO: a collection of workflows to benchmark phylogenetic placement. *Bioinformatics*, 36(21):5264–5266, 2021.

[4] Kevin Liu, C Randal Linder, and Tandy Warnow. RAxML and FastTree: comparing two methods for large-scale maximum likelihood phylogeny estimation. *PloS one*, 6(11):e27731, 2011.

[5] Siavash Mirarab, Nam-phuong Nguyen, Sheng Guo, Li-San Wang, Junhyong Kim, and Tandy Warnow. PASTA: ultra-large multiple sequence alignment for nucleotide and amino-acid sequences. *Journal of Computational Biology*, 22(5):377–386, 2015.

[6] Morgan N Price, Paramvir S Dehal, and Adam P Arkin. Fasttree 2–approximately maximum-likelihood trees for large alignments. *PloS one*, 5(3):e9490, 2010.

[7] Eleanor Wedell, Yirong Cai, and Tandy Warnow. SCAMPP: scaling alignment-based phylogenetic placement to large trees. *IEEE/ACM Transactions on Computational Biology and Bioinformatics*, 2022. doi:10.1109/TCBB.2022.3170386.
